# Autonomous electrochemical system for ammonia oxidation reaction measurements at the International Space Station

**DOI:** 10.1038/s41526-023-00265-4

**Published:** 2023-03-08

**Authors:** Camila Morales-Navas, Roberto A. Martínez-Rodríguez, Francisco J. Vidal-Iglesias, Armando Peña, Joesene J. Soto-Pérez, Pedro Trinidad, José Solla-Gullón, Toshko Tzvetkov, Jonathan Doan, Eugene S. Smotkin, Eduardo Nicolau, Juan M. Feliu, Carlos R. Cabrera

**Affiliations:** 1 Department of Chemistry, University of Puerto Rico, Río Piedras Campus, San Juan, PR 00925-2537 USA; 2 Institute of Electrochemistry, University of Alicante Ap. 99, 03080 Alicante, Spain; 3 Department of Chemistry and Biochemistry, University of Texas at El Paso, El Paso, TX 79968 USA; 4 NuVant Systems, Inc., 130 N. West Street, Crown Point, IN 46307 USA; 5 Department of Chemistry and Chemical Biology, Northeastern University, 360 Huntington Ave., Boston, MA 02115 USA

**Keywords:** Electrocatalysis, Water resources

## Abstract

An autonomous electrochemical system prototype for ammonia oxidation reaction (AOR) measurements was efficiently done inside a 4'' x 4'' x 8'' 2U Nanoracks module at the International Space Station (ISS). This device, the Ammonia Electrooxidation Lab at the ISS (AELISS), included an autonomous electrochemical system that complied with NASA ISS nondisclosure agreements, power, safety, security, size constrain, and material compatibility established for space missions. The integrated autonomous electrochemical system was tested on-ground and deployed to the International Space Station as a “proof-of-concept” ammonia oxidation reaction testing space device. Here are discussed the results of cyclic voltammetry and chronoamperometry measurements done at the ISS with a commercially available channel flow-cell with eight screen-printed electrodes, including Ag quasi-reference (Ag QRE) and carbon counter electrodes. Pt nanocubes in Carbon Vulcan XC-72R were used as the catalyst for the AOR and 2 μL drop of Pt nanocubes/ Carbon Vulcan XC-72R, 20 wt%, ink was placed on the carbon working electrodes and allowed to dry in air. After the AELISS was prepared for launch to the ISS, a 4 days delayed (2 days in the space vehicle Antares and 2 days space transit to the ISS) cause a slight shift on the Ag QRE potential. Nevertheless, the AOR cyclic voltametric peak was observed in the ISS and showed ca. 70% current density decrease due to the buoyancy effect in agreement with previous microgravity experiments done at the zero-g aircraft.

## Introduction

Ammonia (NH_3_) is a small and uncharged molecule typically used as a fertilizer, refrigerant, a fuel, and it is generated in wastewater processes^1^. Since ammonia is a fuel with a high energy density, it is possible to take this advantage by converting ammonia to nitrogen and electrical energy via the ammonia oxidation reaction (AOR). This reaction requires a catalyst to decrease the energy barrier that prevents the molecule from reacting and transforming into nitrogen. The AOR has been taken to the International Space Station (ISS) using an autonomous potentiostat system with electrode arrays, fluid pumps, and liquid reservoirs, and an autonomous potentiostat.

The anodic electrochemical oxidation of ammonia was done on platinum nanocubes^2^ catalyst on screen-printed carbon electrodes (SPE). The cited literature suggests that under standard conditions, the products of the AOR on monocrystalline platinum (i.e., Pt{100}) is molecular nitrogen at an applied bias of 0.65 V vs. NHE. Nevertheless, other oxides of nitrogen may form at more positive potentials^3,4^. The gas molecules produced by the electro-oxidation of ammonia can detach from the catalyst interface due to the buoyancy effects that are exerted when in the presence of gravity. Below you may find an AOR mechanism developed by Gericher-Mauerer mechanism^5^.

1 $$NH_{3(aq)} \to NH_{3ads}$$

2 $$NH_{3ads} + OH^ - \to NH_{2ads} + H_2O + e^ -$$

3 $$NH_{2ads} + OH^ - \to NH_{ads} + H_2O + e^ -$$

4 $$NH_{xads} + NH_{y\;ads} \to N_2H_{x + y\;ads}$$

5 $$N_2H_{x + y\;ads} + \left( {x + y} \right)OH^ - \to N_2 + \left( {{{{\mathrm{x}}}} + {{{\mathrm{y}}}}} \right)H_2O + \left( {x + y} \right)e^ -$$

6 $$NH_{ads} + OH^ - \to N_{ads} + H_2O + e^ -$$

Under microgravity conditions the AOR has shown to have a lower current density because of the lack of buoyancy which allows the gaseous molecules to remain/stay near or at the electrode catalyst interface^6–8^. The lack of buoyancy for mass transfer convection affects the efficiency of the AOR at the platinum surface^6^. In a parabolic flight where a direct ammonia alkaline fuel cell (DAAFC) was used, the performance decreases up to 27% when using platinum nanocubes supported on Vulcan (Pt-V)^8^. This catalyst was selected for the ISS AOR study since it is robust and provides the means to achieve reproducibility in our experiments. In addition, it showed the highest AOR current densities^9^.

The purpose of the Ammonia Electrooxidation Lab at the ISS (AELISS)^10^ project was to develop an autonomous electrochemical systems for studies at the ISS and to validate the previous results under parabolic flights^6–8^ and elucidate the factors affecting the ammonia oxidation reaction during long-term μG conditions at the ISS. There is an interest on electrochemical processes in space for Environmental Control and Life Support System^11,12^.

**Fig. 1:**
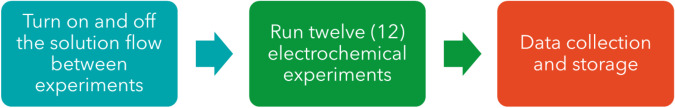
Ammonia Electrooxidation Lab at the ISS electrochemical experimental cycles summary.

For the AELISS experiment an autonomous potentiostat needed to be developed for the ISS, a plug-and-play device. Autonomous potentiostats have been developed for wearable technologies^13^ and smartphones^14^. For the ISS, a 2-U Nanorack (Nanode)^15^ (4'' x 4'' x 8'') was connected to the ISS station equipment rack through a USB-b port. Inside the Nanode the AELISS was placed, which consisted of an autonomous potentiostat, two screen-printed electrode (SPE) Channel Flow-Cells (*Metrohm DropSens)*, two Dolomite Microfluidics peristaltic micropumps, two liquid plastic containers, and a USB flash data storage drive. The autonomous potentiostat, designed and produced by NuVant Systems Inc., controlled all the AELISS components. The AELISS was launched to the ISS on a cargo resupply mission CRS-14/NG-14, in the vehicle Antares, at 9:38 p.m. EDT on October 1, 2020. The data acquisition followed is shown in Fig. 1.

The aim of this research work is to create an autonomous electrochemical device able to improve the time and reproduction of multiple cyclic voltammetry and chronoamperometry experiments at the International Space Station. This will provide a better insight into the selected platinum nanocube catalyst performance for the ammonia oxidation reaction (AOR) and compare results with those generated on Earth gravity.

## Methods

### Pt nanocubes catalyst synthesis

**Fig. 2:**
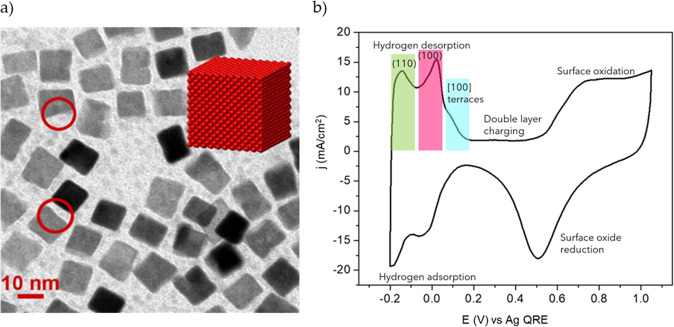
Transmission electron microscopy (TEM) and cyclic voltammetry (CV) of platinum nanocubes prepared by water-in-oil microemulsion in the 25% HCl in the aqueous phase. **a** TEM and **b** CV. (Panel **a** with *JACS* permission^2^).

**Fig. 3:**
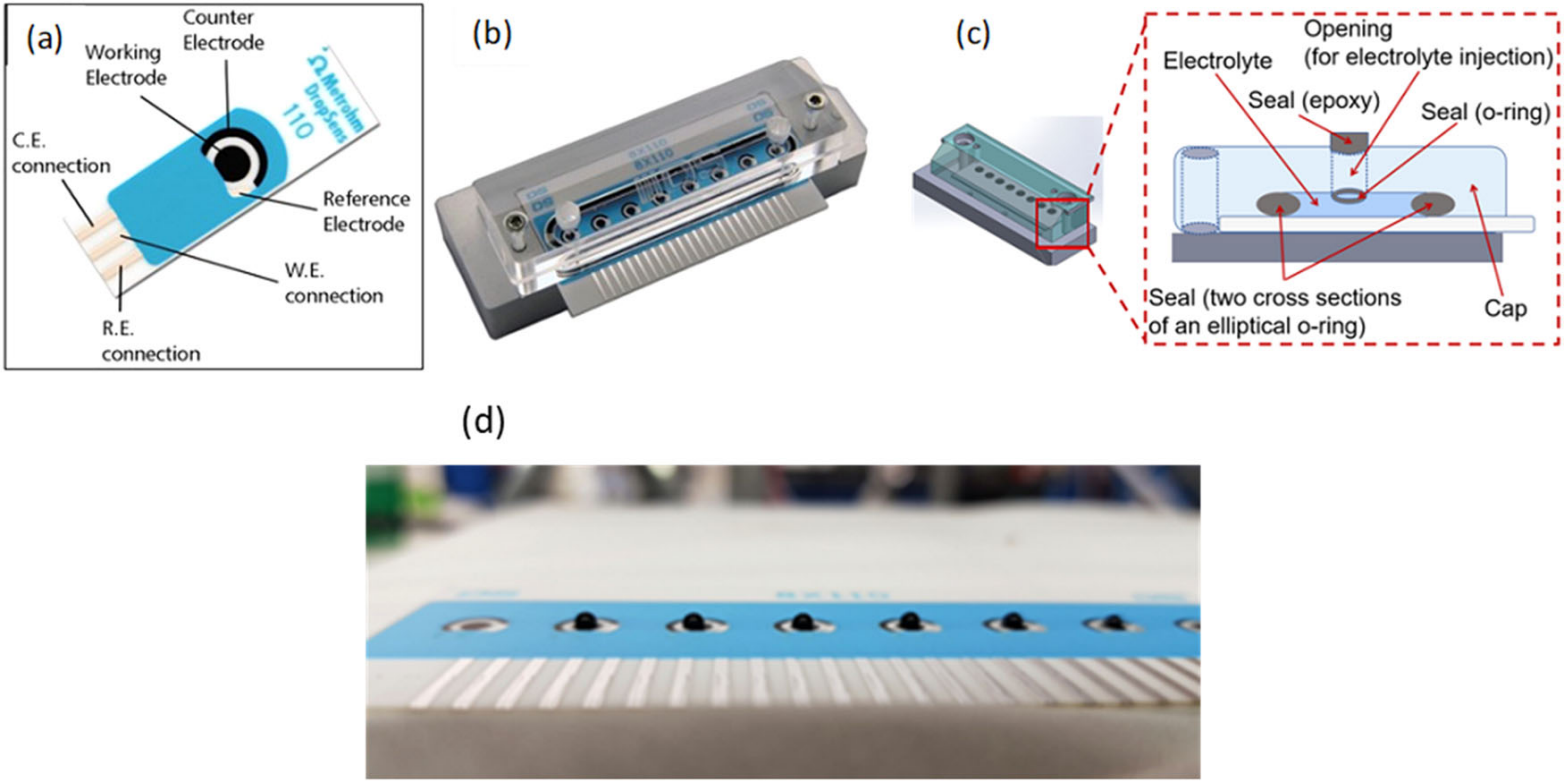
Screen-printed electrodes and DropSens Channel-Flow Cell. **a** Picture of screen-printed carbon electrodes (SPE), **b** Metrohm DropSens Channel Flow-Cell for eight format screen-printed electrodes with reference and counter electrodes used in the AELISS electrochemical system, **c** simplified schematic of the transversal view of a flow-channel to illustrate how the liquid electrolyte will be introduced in the flow channels and how this electrolyte will be contained, and **d** picture of SPE with a 2-μL drop of Pt nanocubes on Carbon Vulcan XC-72R ink.

Electrochemical ammonia oxidation was done on Pt nanocube modified screen-printed electrodes (SPE) from Metrohm. The platinum nanocubes (Pt NC’s) were synthesized by the Water-in-Oil (w/o) Microemulsion method described by R. Rodríguez-Martínez et al. (see Fig. 2)^9^. In brief, Pt nanocube were obtained by reducing 0.1 M H_2_PtCl_6_ in diluted HCl solution with sodium borohydride (NaBH_4_) using w/o microemulsion of water /polyethylene glycol dodecyl ether (BRIJ®30) /n-heptane. Afterward, Pt NC’s were cleaned thoroughly with acetone and deposited over Vulcan XC-72R carbon support in a mass ratio of 20:80. Then, 2 μL of catalyst solution was drop cast on the twelve SPE electrodes and gently dried with ultra-pure Argon (See Fig. 3a). All experiments were performed in 0.05 M (NH_4_)_2_SO_4_ + 0.18 M NaOH solution at pH = 12.9. To prepare this solution first, the 0.18 M NaOH solution was bubbled with Argon for 15 min and afterward the NH_3_ precursor was added. Solution containers were carefully filled with a syringe with a needle, strictly avoiding bubbles to get attached during the process. The containers were sealed 4 days before running the experiments.

### Experiments

Twelve amperometric and cyclic voltammetry experiments were done as a way to assess ammonia electrooxidation events. The screen-printed electrodes (SPE) have a ceramic substrate with 33 × 78 x 1 mm dimensions with the electric contacts made of silver. The electrochemical cell consists of a SPE with 2.56 mm in diameter, a carbon auxiliary electrode, and a quasi-reference silver electrode. The working electrode has a 300 Ω resistance to comply with the counter electrode demand, which is smaller in surface area than the auxiliary electrode. The electrodes were prepared by placing 2 μL drop of Pt nanocubes/ Carbon Vulcan XC-72R ink on the SPE working electrodes. The catalysts’ performance was tested at the International Space Station (ISS) and compared with earth-based measurements. These experiments were conducted inside a Nanoracks 2U described in the hardware section at the International Space Station.

### Hardware description

The AELISS is a miniaturized version of the Electrochemical Microgravity Laboratory (EML) comprised of two major components: the Electronic Rack (ER) and the Electrochemical Equipment Box (EEB)^6–8^.

**Fig. 4:**
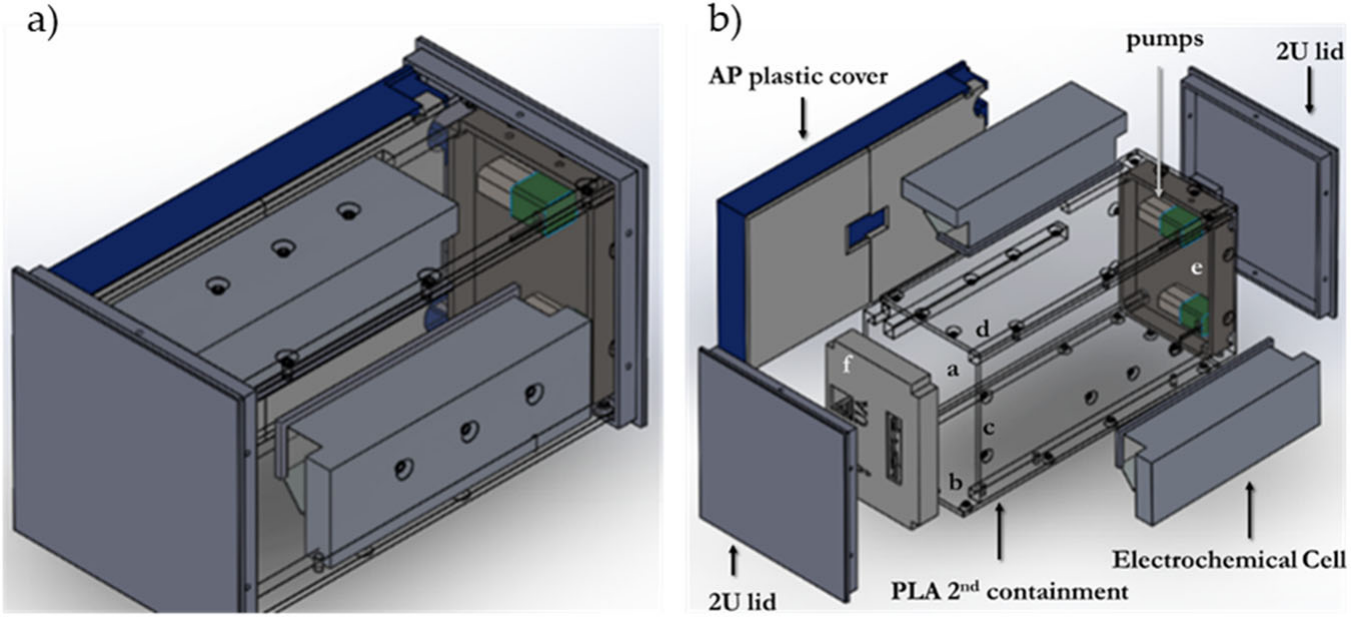
Ammonia Electrooxidation Lab at the ISS (AELISS) device. **a** Illustration of the closed AELISS device and **b** schematic Isometric view of the plastic protective frame with the electrochemical cells. The AELISS had a potentiostat and its plastic cover, plastic protector box frame, two electrochemical cell arrays, two screen-printed electrodes (SPE), two peristaltic pumps, and a storage device.

**Fig. 5:**
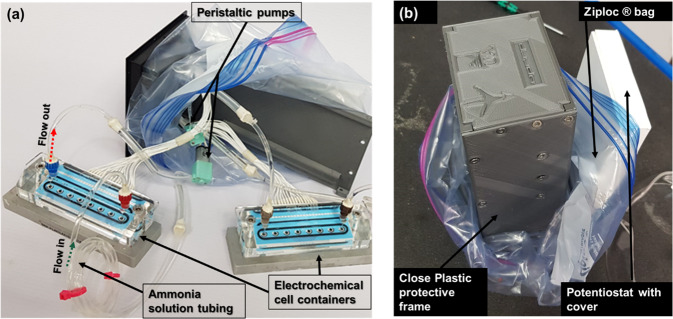
AELISS ammonia solution flow system. **a** Open device picture showing the two sets of closed loops of ammonia solution inside the protective plastic frame and **b** the closed device inside the Ziploc® bag.

Ammonia Electrooxidation Laboratory at the ISS (AELISS) was inside a 2U Nanoracks module (NR-2U) of 4'' x 4'' x 8'' dimensions and connected to the ISS power rack via a USB-b port (5 V and 5 A). AELISS is comprised of two main parts: the electrical module and the electrochemical containers. The electrochemical containers are comprised of two sets of three-electrode electrochemical cell setup. The AP controlled the two peristaltic pumps that pumped the ammonia solution to the electrochemical flow cell and two sets of six (6) electrochemical experiments were run. AP provides a fixed potential to an electrochemical cell setup while it monitors the current as a function of time; this is a chronoamperometry experiment (CA). Afterward, on another electrochemical cell setup, the system controls a changing potential while it monitors the current generated (i.e. cyclic voltammetry (CV)). The electrochemical cell setup consisted of two sets of six screen-printed electrodes shown in Fig. 3. The working electrodes were modified with Platinum Vulcan catalyst ink^9^. The electrochemical part is triply contained in order to avoid any unlikely leakage of diluted ammonia solution (0.05 M (NH_4_)_2_SO_4_ in 0.18 M NaOH) following safety guidelines. This setup holds a maximum of 10 mL 0.05 M Ammonia solution at pH = 12.9. The experimental data was stored on a memory card. Figure 4 illustrates the closed Ammonia Electrooxidation Lab at the ISS (AELISS) device and Fig. 5 shows the open device picture of two sets of closed loops of ammonia solution inside the protective plastic frame and the closed device inside the Ziploc^®^ bag.

### Electronic control device from NuVant Systems Inc

The electronic device contains an autonomous potentiostat, a smart switch, and a storage device. NuVant Systems Inc. provided the in-house modified potentiostat. In the energy power agreement contract between NASA and *Nanoracks*, the required energy power for the autonomous potentiostat system could not exceed the USB 2.0 power, i.e., 5 V and 500 mA^15^. The potentiostat has a PCB board with a single range of 1 mA that can control twelve electrochemical cells. The potentiostat interface has a microcontroller board capable of reading at least twelve analog channels and a motor control circuit to control two fluid pumps at 0.45 ml/min that operate at 3 V and 40 mA. The potentiostat can control voltage from −1 V to +1 V at 1 mA maximum current. The compliance voltage was up to 2 V.

**Fig. 6:**
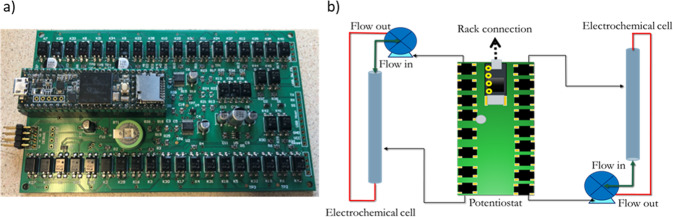
Autonomous potentiostat. **a** NuVant Systems Inc. potentiostat printed circuit board with Teensy microcontroller used in the AELISS electrochemical system and **b** simplified diagram of the AELISS electrochemical system which includes 12 individual electrochemical cells, an autonomous potentiostat, peristaltic pumps (2), and under triple containment.

The *NuVant* components are shown in Fig. 6, which are the following: (1) A Teensy 3.5/3.6 microcontroller board. This family of microcontroller prototype boards had a 32-bit microprocessor with a floating-point unit. It has twenty-five analog inputs connected to 2 ADCs with 13-bit resolution, two analog outputs (DACs) with 12-bit resolution, and >20 DIO. (2) A USB host controller board V2.4 from *Hobby Tronics* was selected as a USB adapter for with preinstalled software/driver for USB flash drive. The USB flash drive was a freshly formatted and completely empty. This is important from security point of view since NASA does not need to warry about virus on the flash drive storage. (3) A two-port USB sharing switch was integrated to redirect the Flash Storage to a Windows software computer automatically. (4) A UGREEN smart switch was modified to allow direct control from the Teensy microcontroller.

### Reporting summary

Further information on research design is available in the Nature Research Reporting Summary linked to this article.

## Results

### Programming

The AELISS system was programmed to turn on 30 min after powering the Nanode containing the AELISS electrochemical system. The Nanode performed twelve experimental runs, turning a peristaltic pump on for 1 min to refresh the contact solution through the SPE before each experiment. Then, the AELISS system performed the potential controlled AOR experiments, cyclic voltammetry and chronoamperometry, to record the current generated that will ultimately describe the ammonia oxidation over platinum nanocubes particles surface on the SPE carbon electrodes at the ISS.

### Cyclic voltammetry

The ammonia oxidation reaction is sensitive to the catalyst facet (i.e., miller indexes), and it is known to occur almost exclusively at the Pt {100} sites. Nevertheless, it could take place between 0.45 V and 0.66 V vs. RHE in polyoriented platinum catalysts. The current peak shoulder observed at 0.55 V is related to the presence of Pt (100) facet domains on the polyoriented platinum catalyst^3^. Although the nanoparticles were preferentially synthesized to form Pt (100) surfaces, there is always the possibility that they agglomerate in the ink solution and block AOR active surfaces.

**Fig. 7:**
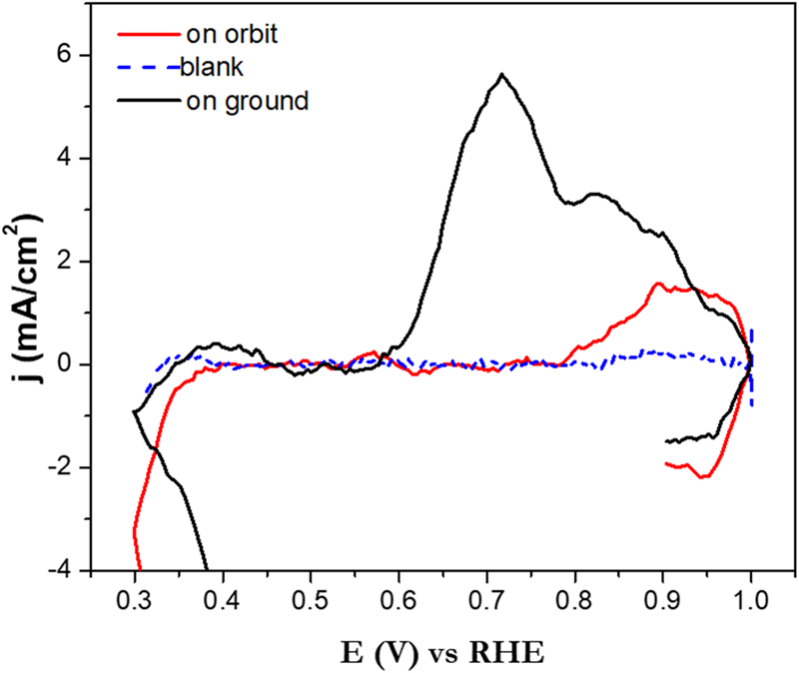
Cyclic voltammetry profiles nanocube Pt nanoparticle-Vulcan electrode in 0.05 M (NH_4_)_2_SO_4_ 0.18 M NaOH solution at pH 12.9 and scan rate of 10 mV/s. Under earth and atmospheric conditions (black line), on-orbit (red line) and 0.18 M NaOH used as blank (blue line).

The AELISS was able to complete the electrochemical experiments successfully. Cyclic voltammetry data was collected and compared with that obtained on-ground before the launching to the ISS (See Fig. 7). Table 1 presents the AOR peak current density generated and % performance reduction on ISS orbit compared to ground conditions. AOR peak current density at 0.7 V vs RHE was used.

**Table 1:** Ammonia oxidation reaction (AOR) peak current density generated and % performance reduction on ISS orbit compared to ground conditions.

AOR peak current density On-ground (mA-cm^−2^)	AOR peak current density On-orbit (mA-cm^−2^)	% AOR performance reduction on ground vs. On-orbit
5.6	1.8	69
6.0	1.6	74

AOR peak current density position at 0.7 V vs RHE was used.

## Discussion

This project and the autonomous electrochemical system have been designed, developed, and tested in the International Space Station. The AELISS system worked adequately and showed that a scale-up autonomous system is possible. The autonomous potentiostat, the micropumps, and the storage device were able to do the ammonia oxidation reaction in two channel flow-cell with eight screen-printed electrodes, including an Ag quasi-reference (AgQRE) and carbon counter electrodes, at the ISS. Nevertheless, the ammonia oxidation peak was slightly shifted to a more positive potential due to the quasi-reference electrode reactivity and potential shift. A post-mortem electrochemical study needs to be done when the AELISS system returns to Earth. Another possible explanation for the potential shift in the AOR peak current may be due to the catalyst poisoning when under prolonged exposure to alkaline solution in the presence of Ag and carbon electrodes. This may lead to a reaction with Pt-NO_x_ species instead of ammonia molecules. In fact, it is not easy to describe an AOR complex stepwise reaction using current available by electrochemical tools and equipment. Due to the limited space available to perform experiments in the ISS, specialized electrochemical tool options are very constrained. Moreover, the proof-of-concept success of the AELISS system opens opportunities to upscale the system to a more complex experimental design related to life support systems and in-situ resource utilization in NASA space missions. Suborbital payload opportunities, e.g., Virgin Galactic or Blue Origin, will be considered in the near future with an astronaut doing the electrochemical measurement in microgravity. This will shorten the time of the experiment.

## Supplementary information

Supplemental Material

Reporting Summary

## Data Availability

The datasets generated and analyzed during the current study are available from the corresponding author on reasonable request.
