# Supplementary material for: Autonomous electrochemical system for ammonia oxidation reaction measurements at the International Space Station
Source: NPJ Microgravity. 2023 Mar 8;9:20. doi: 10.1038/s41526-023-00265-4 (PMC9995563; doi:10.1038/s41526-023-00265-4)
Supplement: Supplementary file 1 — Supplemental Material [file 41526_2023_265_MOESM1_ESM.pdf]

SUPPLEMENTARY INFORMATION

Autonomous Electrochemical System for Ammonia Oxidation Reaction Measurements at the International Space Station

Camila Morales-Navas[1*], Roberto A. Martínez-Rodríguez[1,2], Francisco J. Vidal-Iglesias[2], Armando Peña[3], Joesene Soto[1], Pedro Trinidad[1], José Solla-Gullón[2], Toshko Tzvetkov[4], Jonathan Doan[4], Eugene S. Smotkin[4,5], Eduardo Nicolau[1], Juan M. Feliu[2], and Carlos R. Cabrera[1,3*]

[1]Department of Chemistry, University of Puerto Rico, Río Piedras Campus, San Juan, Puerto Rico 00925-2537

[2]Institute of Electrochemistry, University of Alicante Ap. 99, 03080 Alicante, Spain

[3]Department of Chemistry and Biochemistry, University of Texas at El Paso, El Paso, Texas 79968

[4]NuVant Systems, Inc., 130 N. West Street Crown Point, Indiana 46307

[5]Department of Chemistry and Chemical Biology, Northeastern University, 360 Huntington Ave., Boston, Massachusetts 02115

SUPPLEMENTARY METHODS

Platinum synthesis

Catalysts were synthesized in the laboratory. In particular, platinum nanocubes were synthesized by the water in oil emulsion method presented by Martinez-Rodriguez et al. 11 g of Brij30 and 2.1 mL of chloroplatinic acid 0.1M were added to 38.29 g of n-heptane in a container and the mixture was well mixed. Brij30 is a nonionic surfactant used to disperse the chloroplatinic acid particles by steric stabilization. This step was followed by the addition of 0.079g of sodium borohydride to reduce the dispersed platinum precursor. The reaction took place for 20 minutes. After it was finished, acetone was added to induce the precipitation of the platinum nanocubes. Once precipitated the particles were rinsed with acetone, methanol, and deionized water. Cubic particle formation was induced by adding HCl during the reduction process. The method for making the platinum supported on Vulcan™ nanoparticles is as follows: platinum nanoparticles were reduced with sodium borohydride by the method previously explained, then added 39 mL of a solution of 5mg/L of Vulcan™ in n-Heptane, to make a catalyst with a Pt/Vulcan™ ratio of 20:80. The solution was mixed for 30minutes. The

platinum loading of the Pt in Vulcan™ catalysts was ca. 20% Pt as determined by thermogravimetric analysis.

Pt Nanocube Sample Characterization

The samples chosen were cubic-shaped platinum nanoparticles and cubic shaped platinum deposit over Vulcan XC 72R carbon support in a mass ratio 20:80 from the same synthesis. Transmission electron microscopy (TEM) was done to have insight about the shape, size, and distribution rate of the particles. The instrument used was a JEOL, JEM-2010 working at 200kV and 120kV. The catalyst was dispersed in an isopropanol solution and sonicated for 30s. A small drop of catalyst was deposited in a cooper grid covered by Formvar. More than 200 particles from different places on the grid were analyzed to prepare a particle size histogram (see Figure 1).

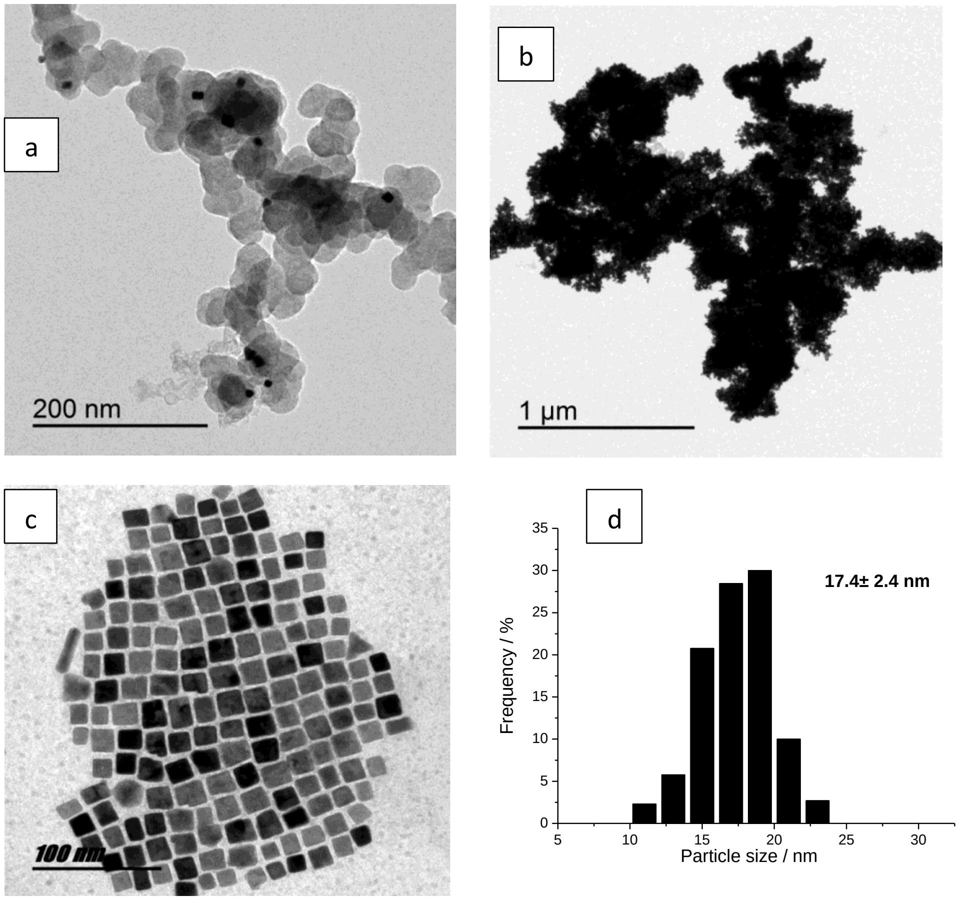

Supplementary Figure 1. (a) Sample: Pt cubes/V 20 wt% (Vulcan XC-72R), (b) Pure Pt in water after cleaning, (c) Pt nanocube sample from the micro-emulsion, (d) TEM images with the corresponding histogram.

Thermogravimetric Analysis

The platinum loading was evaluated by thermogravimetric analysis with a Mettler-Toledo balance, TGA/SDTA851. The temperature range was 10 °C per minute from 25° to 850° in oxidative atmosphere of N_2/O_2= 4:1. The experimental and nominal loading agreed, i.e. 20% (See Figure 2).

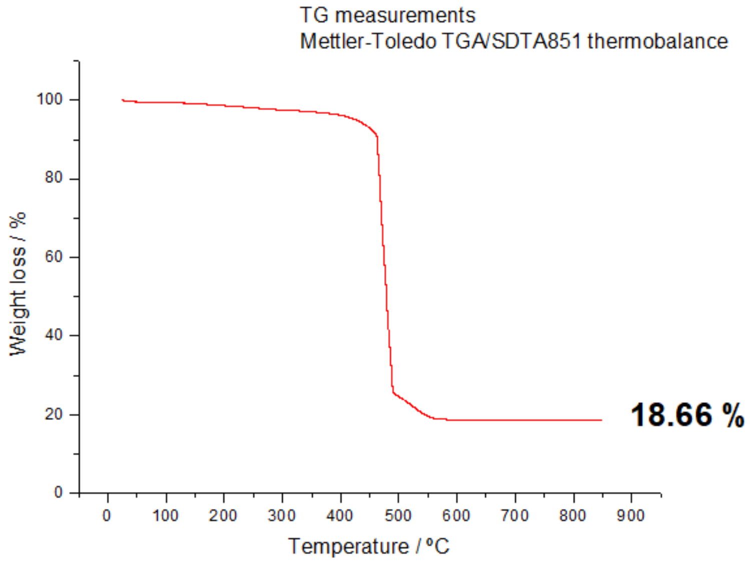

Supplementary Figure 2. Thermogravimetric Analysis (TGA) of Platinum Nanocubes at Vulcan XC-72R sample.

Independent Screen printed electrode evaluation:

The SPE electrode shown in figure 3b is like the in-series SPE used for the ISS experiments. The single SPE electrodes were used as a starting point of the project to test and eventually select the best solution and Pt catalyst for the ammonia oxidation reaction. The figure 3a and 3b show the process of drying with Argon an ink drop of 6μL Pt cubes/V 20 wt% (Vulcan XC-72R). For this study, individuals' SPE were evaluated as shown in figure 4.

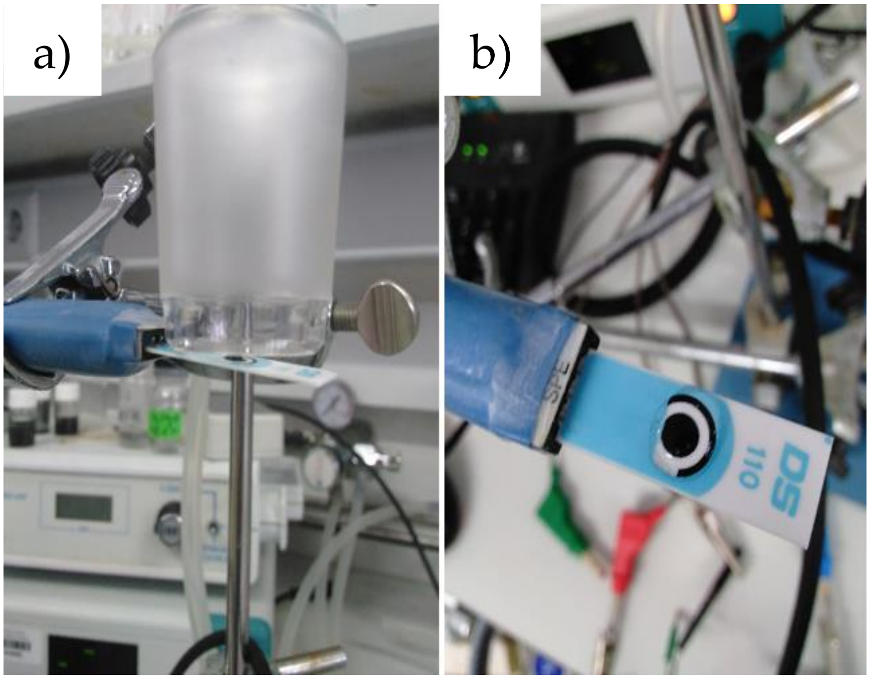

Supplementary Figure 3. a) Dropcasted catalyst onto the individual *Dropsens* microelectrode with an $Ar_{(g)}$ bell and b) Image of deposited catalyst ready for electrochemical analysis.

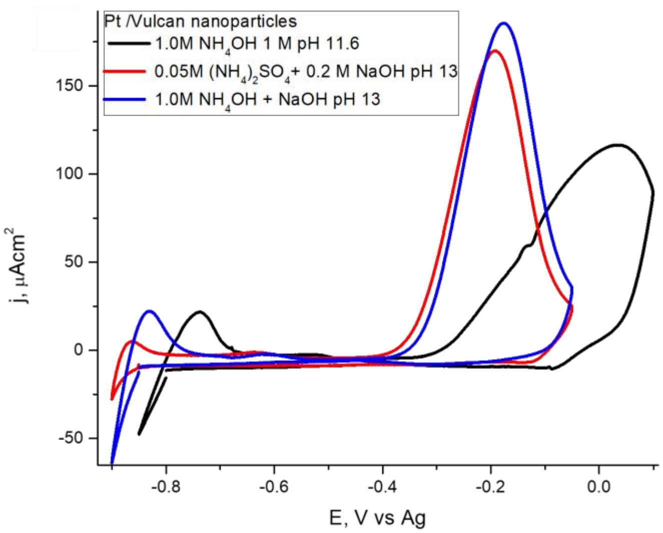

Supplementary Figure 4. Voltammetic profiles of AOR Pt cubes/V 20 wt% (Vulcan XC-72R) in different solutions at a scan rate of 10mVs^{-1}.

The best solution concentration for the catalyst loading and current was 0.05 M $(NH_4)_2SO_4$ in 0.2 NaOH.

Temperature profile :

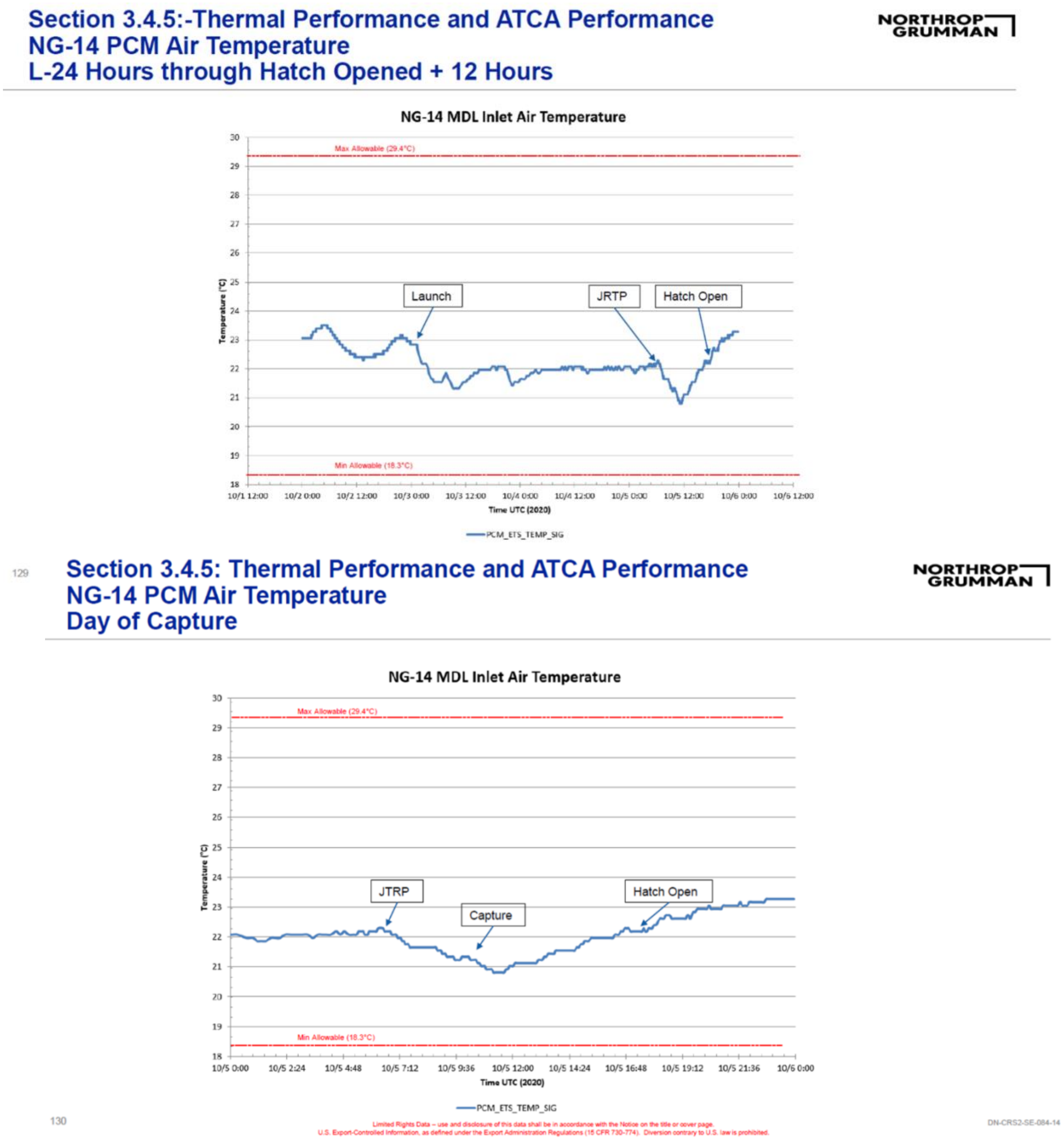

Supplementary Figure 5: The 1st plot shows PCM cabin temperature in degrees Celsius from Final Cargo Load until Hatch open. The 2nd plot is a “zoomed in” section that focuses on capture day.

Electrochemical Cell Array

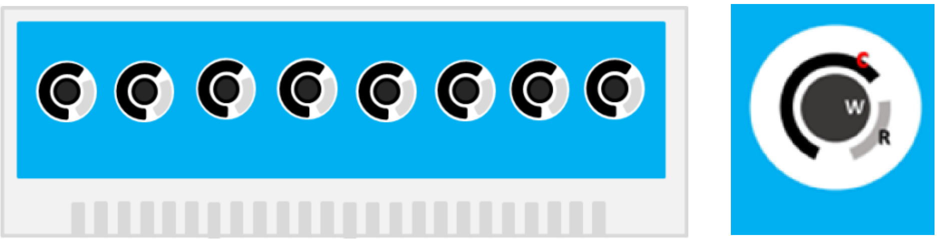

Supplementary Figure 6. Schematic of the electrochemical cell array: C: Counter Electrode, W: Carbon Working Electrode, and R: Ag quasi reference electrode (Ag QRE)

Cyclic Voltammetry

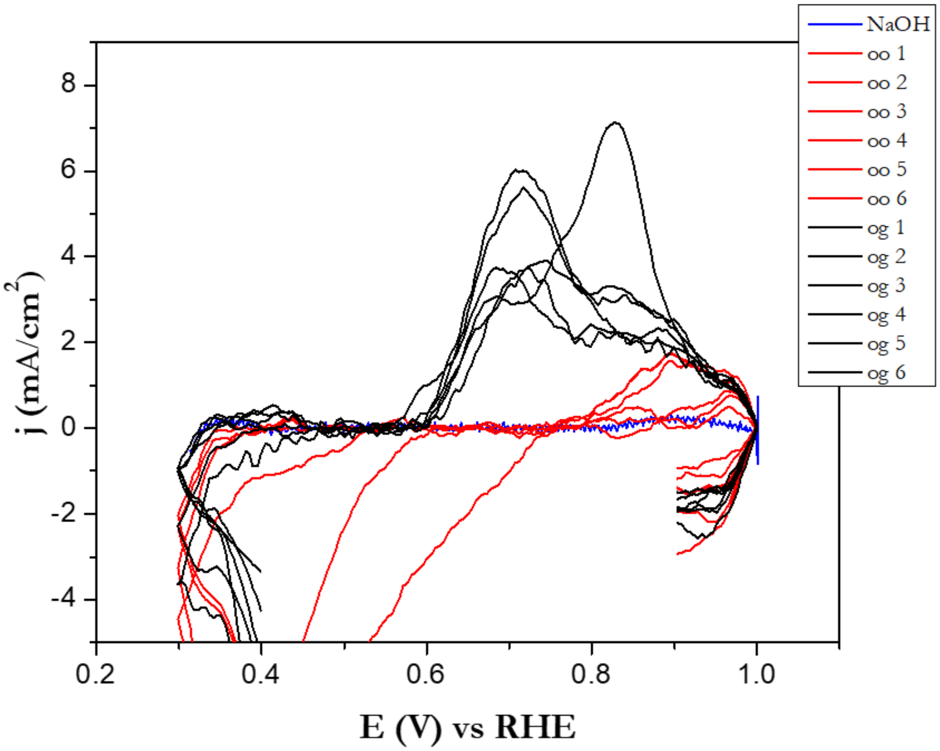

Supplementary Figure 7: Experiments were done in AELISS electrochemical system with Pt-V electrode in 0.05M $(NH_4)_2SO_4$ in 0.18 M NaOH at pH 12.9 at 10 mV/s under normal conditions (black line) and on-orbit at the ISS (red line) and black of 0.18 M NaOH on-ground (blue line).

Supplementary Table 1: Moles of oxidated NH_3

NH_3 oxd moles	active area of Pt (C/cm²)	Peak area (cm²)	Faraday's constant (C/mol)	Avogadro number	NH_3 molecules	peak On orbit
1.42E-10	2.10E-04	0.065	96485	6.02214E+23	8.56E+13	oo1
1.59E-10		0.073			9.57E+13	oo2
7.25E-11		0.033			4.36E+13	oo3
3.53E-10		0.162			2.12E+14	oo4
4.56E-10		0.210			2.75E+14	oo5
3.94E-10		0.181			2.37E+14	oo6
average	**NH_3 molarity**	**NH_3 moles in 9 μL**				
2.63E-10	0.1	9.00E-07				

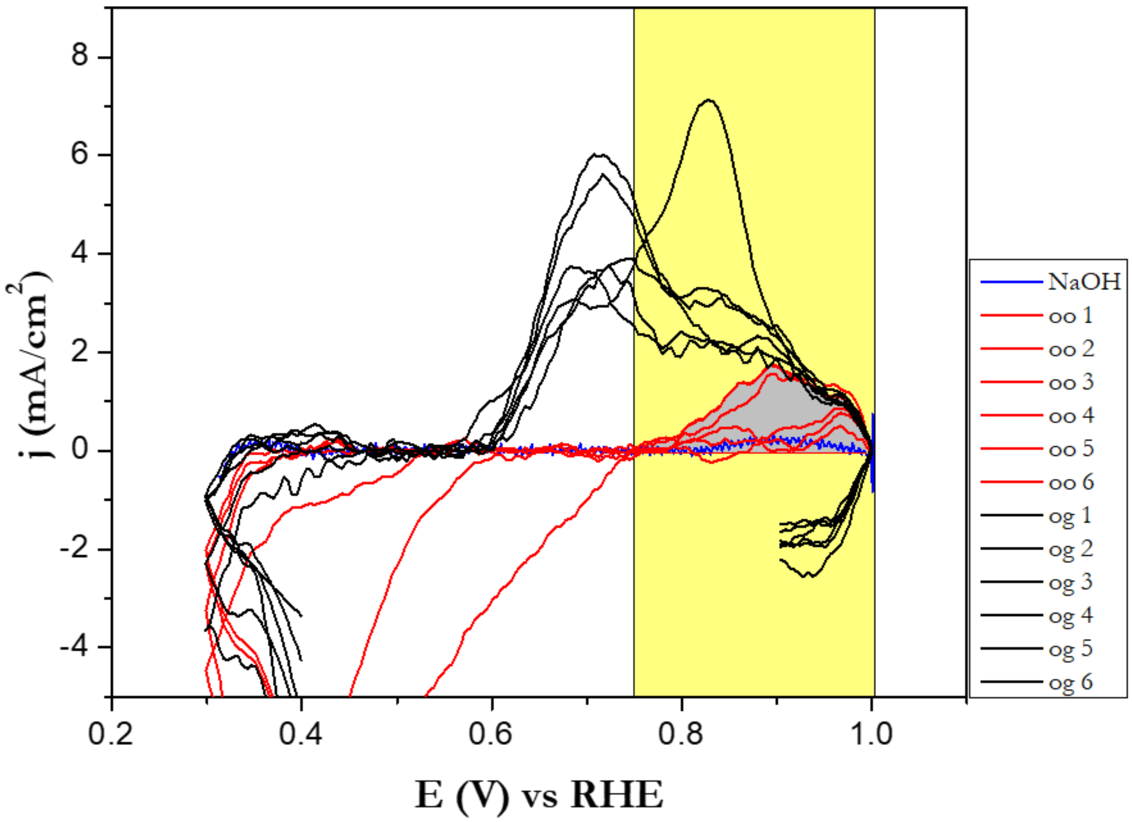

Supplementary Figure 8: The Pt-V electrode in 0.05M $(NH_4)_2SO_4$ in 0.18 M NaOH at pH 12.9 at 10 mV/s. The gray region shows the area used to calculate the average oxidation moles of NH_3 on-orbit at the ISS (red line) shown in table 1.

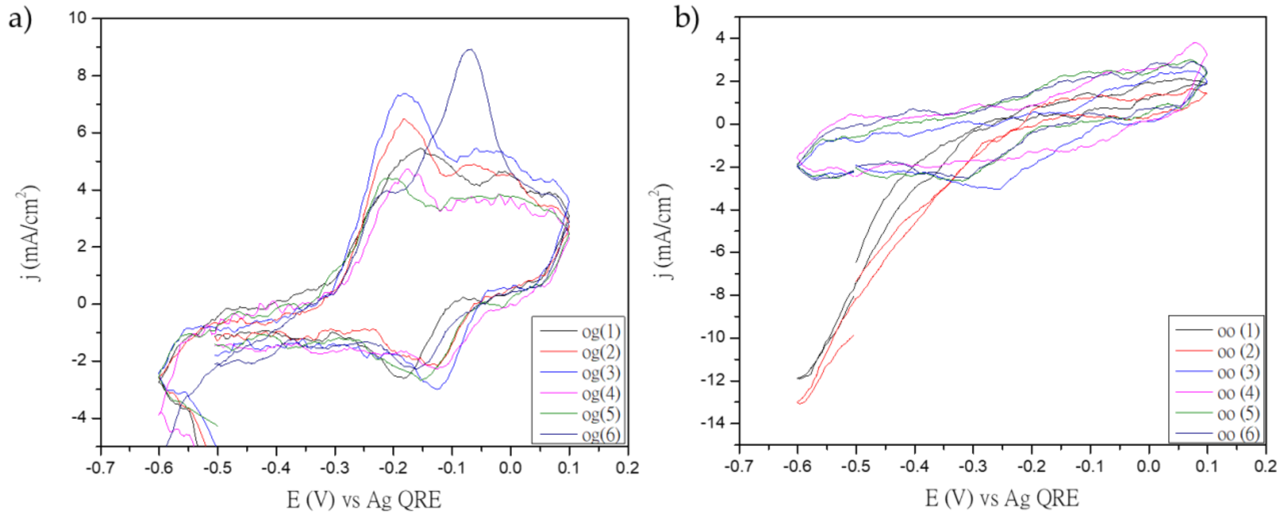

Supplementary Figure 9: Experiments were done in AELISS electrochemical system with Pt-V electrode in 0.05M $(NH_4)_2SO_4$ in 0.18 M NaOH at pH 12.9 at 10 mV/s under a) normal conditions and b) on-orbit at the ISS.

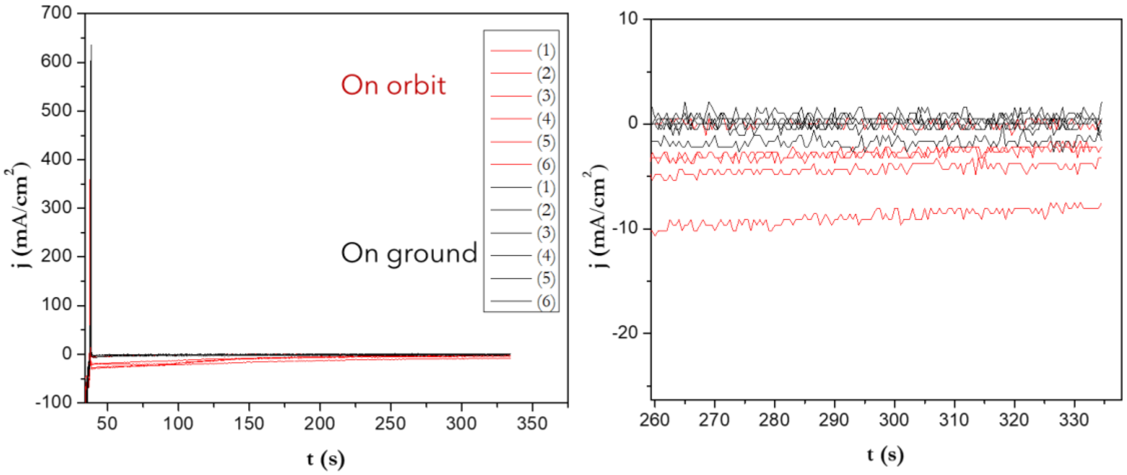

Supplementary Figure 10: Chronoamperometric measurements of a Pt-V electrode in 0.05M $(NH_4)_2SO_4$ in 0.18 M NaOH at 0.6 V vs. RHE under normal conditions (black line) and on-orbit at the ISS (red line).

NuVant **autonomous potentiostat with its main AELISS electrochemical system components**

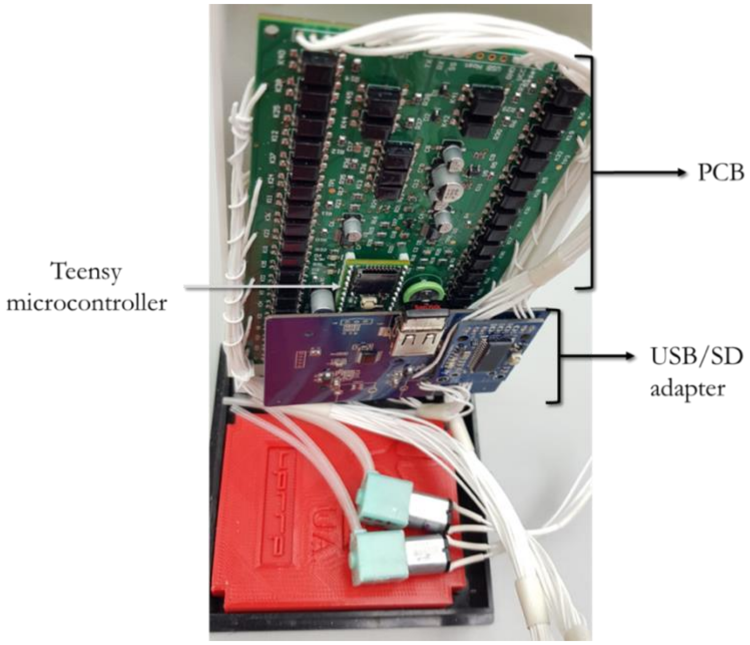

Supplementary Figure 11. Picture of the *NuVant* autonomous potentiostat with its main AELISS electrochemical system components.

Pressure Testing

Payload leak tests are considered adjunctive to payload qualification acceptance tests which are part of the success criteria for NASA approval. The suggested methods to test an "external-to-internal" payload, where the pressure may change, and mass loss may be lost after vacuum exposure, were explored.[1] Pressure testing was done to ensure that the barbed unions and flow bolts could hold the ammonia solution flow without leaking. A summary of the tests performed for each containment is included in **Table 2**. Phenolphthalein was used as a pH indicator since it is colorless at neutral pH and changes color to red-violet at pH higher than 8.[2] The testing procedure was the following: First, the containment was sealed as expected for the final launch, sprayed with the indicator to identify possible leakage. Afterward, it was exposed to a vacuum for 5 minutes inside a non-powered vacuum oven. The tests were done at room temperature.

Supplementary Table 2. Summary of pressure and vacuum results.

**Containment**	**Weight ± 0.1 (g)**	**Pressure** atm (30inHg)	**Vacuum (inHg)**	**Findings**
**1**	Before: 214.8<br>After: 214.7	1	78	No loss of solution or pH change indicator identified
**1 and 2**	Before: 643.0<br>After: 642.9	1	78	No loss of solution or pH change indicator identified
**1, 2 and 3**	Before: 661.7<br>After: 661.6	1	78	No loss of solution or pH change indicator identified

Supplementary Figure 12 shows the primary containment inside a vacuum oven with a white surface to facilitate the tinted liquid monitoring.

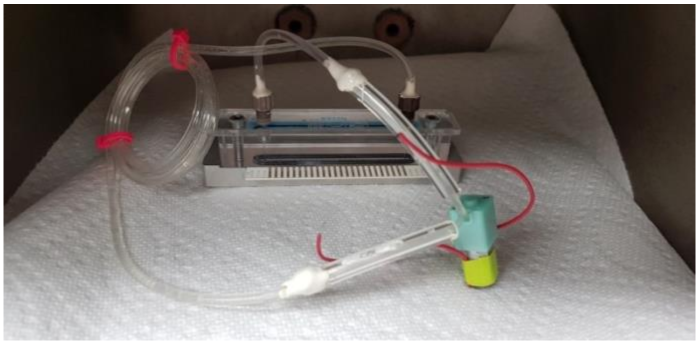

Supplementary Figure 12. Picture of first containment (electrochemical cell with tubing and pump) inside a vacuum oven where the vacuum tests were performed.

SUPPLEMENTARY IMAGES

Assembly process in the laboratory at NASA Wallops Flight Facility

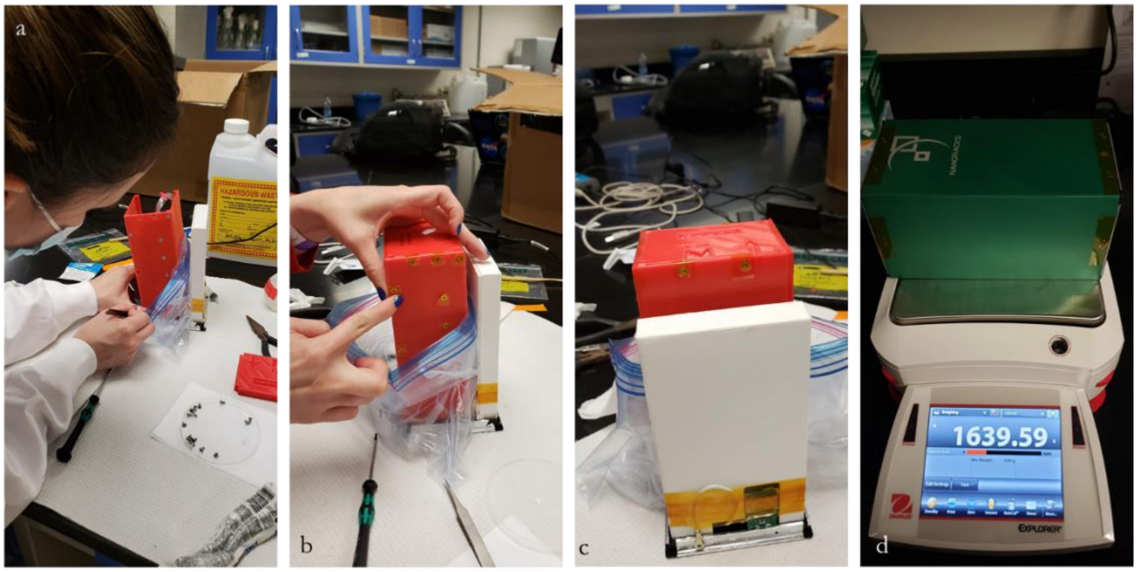

Supplementary Figure 13. Assembly process in the laboratory at NASA Wallops Flight Facility: (a)set screws, (b) insulating with Kapton® tape, (c) device complete, and (d) AELISS electrochemical system inside the 2U Nanoracks box showing device's weight in grams.

SUPPLEMENTARY REFERENCES

1. Lvovsky, O.; Grayson, C. M., Aerospace Payloads Leak Test Methodology. In *American Society for Nondestructive Testing, Fall Conference*, NASA: Houston, TX, United States, 2010.

2. Harris, D. C., *Quantitative Chemical Analysis*. 7th ed.; Craig Bleyer: New York, USA, 2007; p 555.
